# Human-in-the-Loop Modeling and Bilateral Skill Transfer Control of Soft Exoskeleton

**DOI:** 10.3390/s24237845

**Published:** 2024-12-08

**Authors:** Jiajun Xu, Kaizhen Huang, Mengcheng Zhao, Jinfu Liu

**Affiliations:** 1 College of Mechanical and Electrical Engineering, Nanjing University of Aeronautics and Astronautics, Nanjing 210016, China; huangkaizhen@nuaa.edu.cn (K.H.); zhaomengcheng@nuaa.edu.cn (M.Z.); 2 Changzhou Vocational Institute of Industry Technology, Changzhou 213164, China

**Keywords:** soft exoskeleton, adaptive impedance control, reinforcement learning, skill transfer

## Abstract

Soft exoskeletons (exosuits) are expected to provide a comfortable wearing experience and compliant assistance compared with traditional rigid exoskeleton robots. In this paper, an exosuit with twisted string actuators (TSAs) is developed to provide high-strength and variable-stiffness actuation for hemiplegic patients. By formulating the analytic model of the TSA and decoding the human impedance characteristic, the human-exosuit coupled dynamic model is constructed. An adaptive impedance controller is designed to transfer the skills of the patient’s healthy limb (HL) to the bilateral impaired limb (IL) with a mirror training strategy, including the movement trajectory and stiffness profiles. A reinforcement learning (RL) algorithm is proposed to optimize the robotic assistance by adapting the impedance model parameters to the subject’s performance. Experiments are conducted to demonstrate the effectiveness and superiority of the proposed method.

## 1. Introduction

Hemiparesis has gradually become a focal societal concern in recent years. The application of wearable exoskeleton robots has exhibited significant potential to enhance patients’ muscular strength and assist in their daily lives, and rehabilitation is expected to be achieved [1,2,3]. However, most existing rigid exoskeletons are designed with heavy and complicated structures, and the robotic joints are required to align with human biological joints; otherwise, the wearing safety and comfort will be greatly reduced. To address these problems, Harvard University initially introduced the concept of soft exoskeletons, also named “exosuits”; they are a wearable suit-like device used for walking assistance [4,5]. In place of rigid components, soft textile materials are utilized in the lightweight design of the robot, and the driving force is transmitted to the human joints by Bowden cables, which significantly enhances wearing experience and allows wearers to move naturally. Undoubtedly, the advent of soft exoskeletons provides a new solution to assist hemiplegic patients in daily life activities.

Current soft exoskeletons mostly employ cable-driven elements for actuation, and the driving force can be thus smoothly transmitted to the human joints [6,7]. However, due to muscle atrophy and neural unit degeneration of hemiplegic patients, the driving forces provided by present cable-driven exosuits are insufficient to meet their needs when completing a full gait cycle. A variation of the cable-driven actuator is introduced, the twisted string actuator (TSA), where an electric motor axially twists one end of a string to produce linear motion [8]. Compared with traditional cable-driven systems, the main advantages of TSAs lie in their compact structure, direct connection between the motor and tendon, very high reduction ratio, and powerful tendon force, making them suitable for soft exoskeletons. In this regard, high-strength driving force can be generated by the TSAs to motivate the severely impaired patients. Variable stiffness is another important characteristic of the TSA, and its constitutive model has been established in our previous studies to declare the stiffness modulation approach [9]. An exosuit with TSAs is developed to assist elbow motion in daily life activities [10]. A soft knee exosuit is proposed for stair climbing, with TSAs providing high-strength assistance [11]. Although TSAs have been successfully employed in soft exoskeletons, hysteresis and friction phenomena cannot be avoided due to the nonlinear dynamic characteristics of TSAs. Adaptive controllers are expected to be taken into consideration to eliminate modeling uncertainties, especially in the human-exosuit coupled system.

As closely coupled human-centered systems, soft exoskeletons require accurate modeling of the human-robot interaction for appropriate assistance. The nonlinear deformation of flexible textile materials and friction between the exosuit and human skin severely affect the modeling accuracy. For soft robotic mechanisms, model predictive control can achieve closed-loop motion control even with nonlinearities and constraints [12,13]. By taking advantage of this strategy, the modeling uncertainties of the soft exoskeleton can be handled to improve control accuracy and ensure user safety. Kinematic and dynamic models of the human-exosuit coupled system are constructed for ankle assistance, and the exosuit can be adjusted to accommodate to different terrains through impedance learning [14]. A human-exosuit coupled dynamic model is constructed for ankle plantarflexion considering the ankle geometries as well as compliance and friction of Bowden cables [15]. The wearer’s motional and biological signals are both fused to estimate the intention, and an assist-as-needed control strategy is utilized to activate the soft exoskeleton [16]. In addition to traditional posture and electromyogram (EMG) signals that are usually employed in wearable exoskeleton robotics, pattern recognition can be used for labeling and scoring physical exercises performed in front of the Kinect sensor [17]. Wearable sensors can also be employed for robot-assisted stroke rehabilitation by closely conforming to human bodies [18]. A time-independent control system is developed to generate an assistance profile while enabling continuous assistance adjustment to individual walking gait [19]. Although the abovementioned recent studies have addressed the human-exosuit interaction issue with detecting human motion intention, the modeling uncertainties cannot be avoided to simultaneously pursue accurate trajectory and force tracking, and user safety may be even threatened. For hemiplegic patients, one side of their limbs are impaired while the other side remains healthy, and robotic mirror training appears effective for their utilization [20,21]. In present robotic mirror training schemes, the movement trajectories of the healthy limbs (HLs) can be transmitted to the other side, thereby driving the impaired limbs (ILs), but it is not reasonable for complete motion transmission without taking the motor function of the IL into consideration. Essentially, the muscle strength of the IL should be detected and quantified to plan the robotic motion. As a solution to this problem, the concept of skill transfer is a potential means to transfer the HL’s movement capability to the IL [22]. A synergy-based controller is designed for lower limb exoskeletons, so the robotic joint stiffness can be generated in accordance with the subject’s muscular contraction and locomotion behavior [23]. Similarly, for walking assistance for hemiparesis, the movement trajectories and stiffness profiles of the exosuit can be accordingly modulated to fit the patient’s performance. Nonetheless, the formulation of the human-exosuit modeling uncertainty increases the difficulty of obtaining optimal motion and stiffness. Reinforcement learning (RL) algorithm is an effective method that enhances assistance efficiency through continuous human-exosuit interaction. By leveraging RL algorithms to optimize the human-robot coupled model and design the controller, it is expected to exhibit the self-learning capabilities of the exosuit for various patients.

In this paper, an RL-based control strategy is proposed for the exosuit by skill transfer, and the main contributions are listed as follows:

(1) A soft exoskeleton with TSAs is designed and implemented, and the human-exosuit coupled dynamic model is established.
(2) An adaptive impedance controller is proposed for mirror training of hemiplegic patients through transferring the HL’s skill to the IL, and a RL algorithm is developed to optimize the impedance model parameters based on the patients’ movement capability.
(3) Prototype experiments are carried out to validate the feasibility of the proposed robotic system, and its superiority is exhibited through comparison with state-of-the-art methods.

The remainder of this paper is organized as follows: The structure of the exoskeleton system and the human-exosuit coupled model are presented in Section 2. The RL-based adaptive impedance control is put forward in Section 3. The experiments are carried out in Section 4. Finally, this paper is concluded in Section 5.

## 2. System Description

In this section, a soft exoskeleton (exosuit) is designed and implemented to assist walking for hemiplegic patients. The exosuit is driven by TSAs with powerful and variable-stiffness robotic actuation. The mechanical structure and analytic model of the TSA is explained in this section. As the soft exoskeleton is tightly coupled with the human body, the human-robot coupled dynamic model is formulated to clarify the human-exosuit interaction relationship and the regulation law of the actuation stiffness.

**Figure 1:**
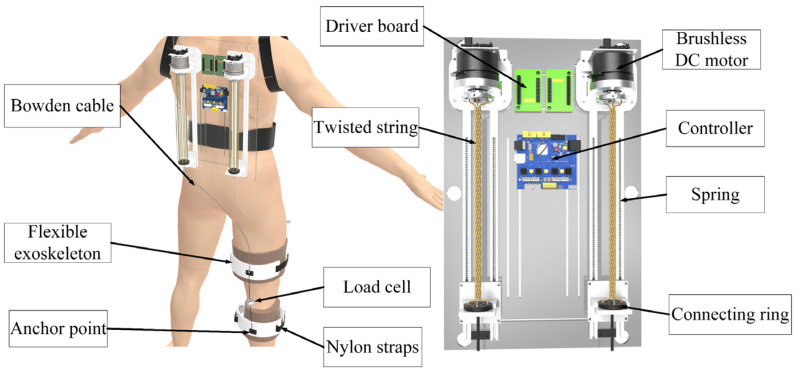
Design scheme of the soft exoskeleton.

A soft exoskeleton is designed to provide hemiplegic patients with knee joint assistance, and its design scheme is presented in Figure 1. TSAs are developed to transmit actuation force to the wearer’s knee joint. The TSA is composed of a motor, a twisted string, and a Bowden cable. When the motor works, the rotation of the motor causes multiple strands to be twisted with each other to constitute the twisted string. As the Bowden cable consists of an outer cable sheath and an inner steel wire, the tendon force is then used to drive the steel wire through a connecting ring. An upper and lower exoskeleton is used to brace the wearer’s thigh and shank with Nylon straps, respectively. The cable sheath is fixed on the upper flexible exoskeleton, and the distal end of the steel wire is connected to the anchor point at the lower flexible exoskeleton. Therefore, once the TSA works and the Bowden cable is tensed, the contracting force between the upper and lower flexible exoskeleton is exerted to provide assistance torque on the knee joint. Generally, two TSAs work collaboratively to drive the flexion and extension of the knee joint. A spring is installed inside the sliding track, storing and releasing the energy during the untwisting process to counteract the hysteresis. The TSAs, along with driver boards and a controller, are equipped at the backpack of the wearer. More details of the exosuit design can refer to the previous studies [9]. The TSAs are equipped at the backpack of the wearer. A load cell is connected in series with the Bowden cable to measure the tension force. Importantly, in response to the specific requirements of various patients, the TSA can modify its stiffness to provide appropriate assistance.

**Figure 2:**
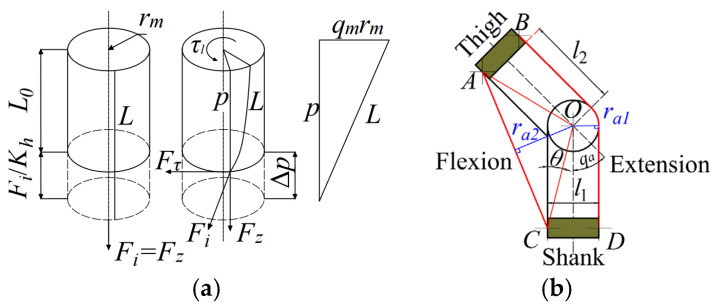
The schematic view of the TSA. (**a**) Geometry of the string during the twisting; (**b**) The schematics of the tendons routing on the knee.

As the TSA is driven from the motor power, the output torque of the motor can be formulated as:

(1) $$\tau_{m}=I_{m}{\ddot{q}}_{m}+V_{m}{\dot{q}}_{m}+\tau_{l}$$

where ${q_{m}=\left[q_{m1},q_{m2}\right]}^{T}\in R^{2}$ represents the motor angular position vector, $I_{m}=diag\left[I_{m1},\;I_{m2}\right]\in R^{2\times 2}$ represents the motor shafts’ inertia moment matrix, $V_{m}=diag\left[V_{m1},\;V_{m2}\right]\in R^{2\times 2}$ represents the motor shafts’ viscous friction coefficient matrix, and $\tau_{l}={\left[\tau_{l1},\;\tau_{l2}\right]}^{T}\in R^{2}$ represents the motor shafts’ load torque vector and can be treated as the input tension of the twisted string. The motor torque is denoted as $\tau_{m}={\left[\tau_{m1},\;\tau_{m2}\right]}^{T}\in R^{2}$, and it yields the input saturation as $\left|\tau_{m}\right|\leq S_{max}$, where $S_{max}$ is the upper bounded value of the motor torque. Consequently, the TSA’s strands connect to the motor shaft, transferring the rotation motion to the linear traction. The analytic model of the twisted string can be represented as:

(2) $$\tau_{l}=\frac{q_{m}r_{m}^{2}}{p}F_{h}$$

where $F_{h}={\left[F_{h1},F_{h2}\right]}^{T}$ is the tension force of the twisted string, p is the length of the twisted string transmission system, and $r_{m}$ is the radius of the twisted string transmission system. The geometric scheme of the twisted string can be seen in Figure 2a. The tension force of the twisted string is highly correlated to each strand constituting the TSA, and it can be formulated as:

(3) $$F_{h}=nF_{i}=\frac{nK_{h}p}{L_{0}}\left(1-\frac{L_{0}}{L}\right)$$

where n is the number of the strands, $K_{h}$ is the strand stiffness, $L_{0}$ is the unloaded length of the strand, and $L=\sqrt{p^{2}+q_{m}^{2}r_{m}^{2}}$ is the loaded strand length. It can be observed from (3) that the stiffness of the TSA can be changed by manipulating the structure of the TSA to accommodate the assistance requirements of various wearers.

The tension force of the twisted string $F_{h}$ is then transmitted to the steel wire of the Bowden cable to motivate the knee motion. A pair of TSAs are used to drive the flexion and extension of the knee joint, respectively, and the schematic view of the TSAs routing at the knee is shown in Figure 2. The Bowden cable output force $F_{h}$ is exerted on the anchor at the flexible exoskeleton, and the knee joint torque can be expressed as:

(4) $$\tau_{a}=R_{a}F_{h}$$

where $R_{a}=\mathrm{diag}\left[r_{a1},r_{a2}\right]\in R^{2\times 2}$ denotes the vertical distance between the knee joint centers and Bowden cable. The distance $R_{a}$ on both sides of the knee joint can be expressed as:

$$r_{ai}=\left\{\begin{array}{c} \lambda \sin\left.(\frac{q_{a}}{2}+\theta \right.),\;\mathrm{Flexion}\;\mathrm{side}\\ r_{e},\;\mathrm{Extension}\;\mathrm{side} \end{array}\right.$$

where $\theta =arctan\frac{l_{1}}{2l_{2}}$, $\lambda =\sqrt{{l_{2}}^{2}+\frac{1}{4}{l_{1}}^{2}}$, $l_{1}$ is the width of the thigh and shank, $l_{2}$ is the distance between the anchor point and knee joint center, and $q_{a}$ represents the knee joint angular position. It should be noted that the assistive torque $\tau_{a}$ is highly correlated with the TSA structure in (3) and the motor power in (1), and the actuation stiffness can be modulated by manipulating the strand length, strand number, material, motor velocity, and torque. In this regard, variable actuation can be provided to accommodate the requirements of patients with different movement capabilities.

Combining (1)–(4), one can obtain the human-exosuit model as:

(5) $$\tau_{m}=I_{m}{\ddot{q}}_{m}+V_{m}{\dot{q}}_{m}+\frac{q_{m}R_{m}^{2}}{p}\left.\left(R_{a}^{-1}\tau_{a}\right)\right.$$

On the other hand, the human knee impedance can be expressed as an impedance model incorporating inertia, damping, and stiffness, i.e.:

(6) $$\tau_{a}=I_{a}{\ddot{q}}_{a}+D_{a}{\dot{q}}_{a}+K_{a}q_{a}$$

where $I_{a}$ is the inertia of the human shank; $D_{a}$ and $K_{a}$ are the damping and stiffness matrices of the knee joint, respectively; $q_{a}$ is the knee joint angular position; and ${\dot{q}}_{a}$ and ${\ddot{q}}_{a}$ are the knee joint angular velocity and acceleration, respectively.

Specifically, joint stiffness and damping are key representative factors for the muscle motor function, which can also be detected by skin surface Electromyogram (EMG) signals. An EMG-driven musculoskeletal model can be established to estimate the joint stiffness $K_{a}$, which constitutes an EMG-stiffness model, and the model parameters can be optimized by combining the theoretical modeling and biomechanical simulation [24]. After that, the joint damping can be accordingly obtained as $D_{a}=v_{a}\sqrt{K_{a}}$, where $v_{a}$ is a constant that can be determined with the AnyBody Modeling System [25,26].

Substituting the human knee impedance model (6) into the human-exosuit coupled dynamic model (5), one can obtain:

(7) $$\tau_{m}=I_{m}{\ddot{q}}_{m}+V_{m}{\dot{q}}_{m}+\frac{q_{m}R_{m}^{2}}{p}\left.\left(R_{a}^{-1}(I_{a}{\ddot{q}}_{a}+D_{a}{\dot{q}}_{a}+K_{a}q_{a})\right)\right.$$

As the human impedance is required to be compliant with the TSA actuation during the operation, the regulation of the TSA stiffness is transferred to the adaptation of the human knee impedance to the specific interaction tasks.

Additionally, when the motor rotates with $q_{m}$, the linear displacement of the TSA can be calculated as $∆X=L-p=\sqrt{p^{2}+q_{m}^{2}r_{m}^{2}}-p$, and the knee joint moves the angle of $q_{a}=∆X/r_{ai}$. Hence, the relationship between the motor angle and knee joint angle can be calculated as:

(8) $$q_{a}=\frac{1}{r_{ai}}(\sqrt{p^{2}+q_{m}^{2}r_{m}^{2}}-p)$$

where the displacement of the TSA should correspond with the distance at the appropriate (flexion or extension) side. For simplicity, (8) can be written as $q_{a}=H(q_{m})$. Accordingly, the angular velocity and acceleration relationship can be derived as ${\dot{q}}_{a}=J\left(q_{a}\right){\dot{q}}_{m}$ and ${\ddot{q}}_{a}=\dot{J}\left(q_{a},{\dot{q}}_{a}\right){\dot{q}}_{m}+J(q_{a}){\ddot{q}}_{m}$.

In this way, the dynamic model of the human-exosuit coupled system is mathematically constructed, where the human physical and biological information are incorporated, and it lays the foundation for the robotic control strategy design.

## 3. Control Strategy

**Figure 3:**
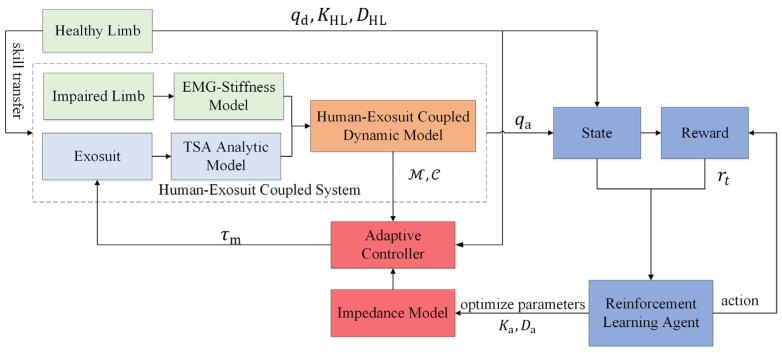
Control diagram.

The proposed exosuit is a typical human-robot coupled system that requires highly coordinated movements with the human body, thereby improving wearing comfort and assistance effectiveness. To obtain the optimal assistance strategy of the exosuit for hemiplegic patients, an adaptive impedance control with RL is proposed in a dual-loop structure, and the control diagram is illustrated as in Figure 3. An impedance controller is designed at the inner loop to drive the robotic joint position to track the desired trajectory while considering the subjective performance. A concept of mirror training is introduced herein, and the target of the inner-loop controller is to make the IL to follow the HL’s motion. At the outer-loop, an RL-based strategy is proposed to optimize the robotic assistance while minimizing the wearer’s voluntary effort, and the impedance parameters are optimized by employing an RL algorithm.

To analyze the control strategy of the robotic system, the human-exosuit coupled model can be rewritten as:

(9) $$\tau_{m}-\Upsilon \left(q_{a},{\dot{q}}_{a},{\ddot{q}}_{a}\right)=M\left(q_{a}\right){\ddot{q}}_{a}+C(q_{a},{\dot{q}}_{a}){\dot{q}}_{a}$$

where $\Upsilon \left(q_{a},{\dot{q}}_{a},{\ddot{q}}_{a}\right)=R_{a}^{-1}I_{a}{\ddot{q}}_{a}+R_{a}^{-1}D_{a}{\dot{q}}_{a}+R_{a}^{-1}K_{a}q_{a}$, $M\left(q_{a}\right)=I_{m}J^{-1}(q_{a})$, $C\left(q_{a},{\dot{q}}_{a}\right)=J^{-1}\left(q_{a}\right)\left(V_{m}-I_{m}\dot{J}\left(q_{a},{\dot{q}}_{a}\right)J^{-1}\left(q_{a}\right)\right)$. The initial trajectory position, velocity, and acceleration are set as zero, i.e., $q_{a}\left(0\right)=0$, ${\dot{q}}_{a}\left(0\right)=0$, and ${\ddot{q}}_{a}\left(0\right)=0$, respectively. The differential terms are upper bounded and defined as $\left|{\dot{q}}_{a}\right|\leq \bar{v}$ and $\left|{\ddot{q}}_{a}\right|\leq \bar{a}$, where $\bar{v}$ and $\bar{a}$ are positive constants. In practical applications, the knee joint velocity and acceleration can be directly measured by inertia measurement units. Some properties of model (9) are given as follows:

***Property 1***: The matrix $M(q_{a})$ is positive definite symmetric and bounded, and its inverse matrix exists.

***Property 2***: The matrix $M\left(q_{a}\right)-2C(q_{a},{\dot{q}}_{a})$ is skew-symmetric.

***Property 3***: The right side of (9) can be written in a linear form as $M\left(q_{a}\right){\ddot{q}}_{a}+C\left(q_{a},{\dot{q}}_{a}\right){\dot{q}}_{a}=W(q_{a},{\dot{q}}_{a},{\ddot{q}}_{a})\theta_{d}$, where the matrix $W(q_{a},{\dot{q}}_{a},{\ddot{q}}_{a})$ is a known function and $\theta_{d}$ is a constant containing physical system parameters.

The basic control objective is to drive the exosuit with tracking the desired trajectory. In this study, as the mirror training strategy is employed to implement the controller, the actual trajectory is assumed as the IL’s movement, and the desired trajectory is commanded by the HL. The error dynamics of the human-exosuit system can be defined as:

(10) $$e=q_{a}-q_{d}$$

(11) $$s=\dot{e}+K_{1}e$$

where $q_{d}$ is the desired trajectory of the knee joint, e is the trajectory tracking error, s is the sliding mode tracking error, and $K_{1}$ is a positive constant.

Then, the controller of the robot system is designed as:

(12) $$\tau_{m}=W(q_{a},{\dot{q}}_{a},{\ddot{q}}_{a}){\hat{\theta }}_{d}+\Upsilon \left(q_{a},{\dot{q}}_{d},{\ddot{q}}_{d}\right)+K_{2}s++K_{3}sgn(s)$$

where $K_{2}$ and $K_{3}$ are control gains, ${\hat{\theta }}_{d}$ is the estimation of $\theta_{d}$, and sgn(·) is the sign function, i.e., $sgn\left(s\right)=\frac{s}{\left\|s\right\|}$, which is set to guarantee the robustness of the control system.

To verify the stability of the control system, a Lyapunov function candidate is formulated as:

(13) $$V=\frac{1}{2}sMs+\frac{1}{2}{\tilde{\theta }}_{d}^{T}\Gamma_{d}^{-1}{\tilde{\theta }}_{d}$$

where ${\tilde{\theta }}_{d}=\theta_{d}-{\hat{\theta }}_{d}$, and $\Gamma_{d}$ is a symmetric positive definite matrix. According to ***Property 2***, the time-derivative of the Lyapunov candidate is derived as:

(14) $$\dot{V}=-s^{T}Ms+s^{T}W{\tilde{\theta }}_{d}+{\dot{\hat{\theta }}}_{d}^{T}\Gamma_{d}^{-1}{\tilde{\theta }}_{d}$$

An adaption law is designed as:

(15) $${\dot{\hat{\theta }}}_{d}=-\Gamma_{d}W^{T}s$$

So, the time-derivative of the Lyapunov function becomes:

(16) $$\dot{V}=-s^{T}Ms$$

As the matrix $M(q_{a})$ and the adaptive gain $\Gamma_{d}$ are positive definite, the Lyapunov candidate function is positive definite, and its time-derivative is negative definite, i.e., $V\left(t\right)>0$, $\dot{V}\left(t\right)<0$. It implies that the tracking error converges to zero, i.e., $q_{a}\to q_{d}$ and ${\dot{q}}_{a}\to {\dot{q}}_{d}$ as t→∞, and the system stability is thus satisfied.

In the controller (12), although the parameter adaption law has been introduced to eliminate the modeling uncertainties, the determination of the appropriate human impedance parameters is important to design the controller. In detail, the human knee impedance ($K_{a}$ and $D_{a}$) should be optimized to simultaneously minimize the trajectory tracking error and facilitate optimization efficiency, which will be elaborated later.

The proposed controller is required to assess the wearer’s physical effort, and necessary assistance intensity should be provided to assist subjects in completing specific walking tasks. For our objective, the robot can achieve the optimal assistance strategy through repetitive learning and exploration, and a learning-based approach is put forward to update the impedance parameters, i.e., $K_{a}$ and $D_{a}$. The procedure of obtaining subjects’ knee impedance through EMG signals involves great uncertainties. Also, it is not helpful to mimic the initial knee impedance by the TSA to complete the assistance because the human motor function cannot be improved in this way. Although the human-exosuit coupled dynamic model has been presented in (9), it is not confident enough to clarify all the model parameters, and the introduction of the adaption law still cannot address the model uncertainties thoroughly. Also, the human impedance parameters vary greatly throughout the operation procedure, and the online update of these parameters cannot be realized with the EMG signals or an EMG-driven model. In this study, an RL algorithm is developed to optimize the human impedance parameters through the exploration of the human-exosuit coupled system in the interaction task.

As the mirror training is introduced into the operation of the exosuit, the physical and biological information on the healthy side can be deployed to optimize the impaired side. The IL is expected to follow the HL’s motion with the assistance of the exosuit, so that the training safety can be autonomously mastered by the user him/herself. In addition, the TSA’s stiffness should mimic the HL’s stiffness to ensure that the robotic motion is compliant enough for the IL’s natural walking. The RL agent receives the kinematics of the robot and both limbs along with main functional muscle EMG signals for training, and the learning target is to maximize the reward function. The immediate reward function is designed with the following rules:

***Rule 1***: Minimize the trajectory tracking error between the IL and HL to ensure the IL can be autonomously mastered and track the motion of the HL, thus ensuring user’s training safety.

***Rule 2***: Mimic the HL’s knee impedance for the IL with the assistance effect of the exosuit, so that the IL’s impedance should be increased and approach the HL’s.

***Rule 3***: Make the generated trajectory smooth enough, avoiding abrupt robotic motion or excessive interactive collision that may harm the IL

Summarizing these rules, the immediate reward function is designed as:

(17) $$r_{t}=\Lambda_{1}{\left(q_{a}-q_{d}\right)}^{2}+\Lambda_{2}{\left({\dot{q}}_{a}-{\dot{q}}_{d}\right)}^{2}-\Lambda_{3}{\left(K_{a}-K_{HL}\right)}^{2}+\Lambda_{4}{\left(D_{a}-D_{HL}\right)}^{2}-\Lambda_{5}{\left({\ddot{q}}_{a}\right)}^{2}$$

where $K_{HL}$ and $D_{HL}$ are the knee joint stiffness and damping of the HL, respectively; ${\ddot{q}}_{a}$ is the knee joint acceleration of the IL; and $\Lambda_{i}$ (i=1,2,…,5) are the weights of different terms, including the joint position, velocity, stiffness, damping, and acceleration, respectively. These weights can be identified with the relative entropy inverse RL algorithms [27].

In this study, a twin delayed deep deterministic policy gradient (TD3) algorithm is employed in the control framework, especially due to its capability of addressing the human-exosuit interaction in continuous action space with a rapid and stable learning performance [28]. Different from traditional critic-actor strategies, the TD3 algorithm deploys double critic networks, and the smaller value is selected to determine the target value. Specifically, the two critic networks have identical architectures, and they are formulated with two different parameters $\theta_{1}$ and $\theta_{2}$. The actor network is parameterized with ϕ. Another target network containing one actor and two critics is also constructed, which is used to compare the real-time reward with the target values. The pseudocode of the RL algorithm is explained in Algorithm 1. During the learning procedure, the RL agent observes the state ($s_{t}$) of the human-exosuit coupled system, including the kinematic and EMG information, and appropriate action ($a_{t}$), i.e., the impedance parameters $K_{a}$ and $D_{a}$, is taken to maximize the reward ($r_{t}$). The critic network is optimized by minimizing the loss function (19), and the target value is defined as in (18). Subsequently, the target network is updated as in (20) with κ denoting the learning rate, and then the learning proceeds into the next iteration. The learning process iterates until the reward is converged and reaches the threshold value $R_{d}$.
**Algorithm 1**: Impedance learning with TD3 algorithm**Initialize:** critic networks $Q_{\theta_{1}}$, $Q_{\theta_{2}}$, and actor network $\pi_{\phi }$ with random parameters $\theta_{1}$, $\theta_{2}$, ϕ
**Initialize:** target networks $\theta_{1}^{\prime }\leftarrow \theta_{1}$, $\theta_{2}^{\prime }\leftarrow \theta_{2}$, $\phi^{\prime }\leftarrow \phi$
**Initialize:** replay buffer B, learning rate κ**For** episode p=1 **to** P **do**
**For** timestep *t =* 1 **to** T **do**

Receive the state $s_{t}$ from the human-exosuit coupled system, select the action $a_{t}$ with exploration noise as $a_{t}=\pi_{\phi }\left(s_{t}\right)+\epsilon$, where $\epsilon \sim N\left(0,\sigma \right)$, calculate the reward $r_{t}$, and receive a new state $s^{\prime }$.

Store transition tuple ($s,a,r,s^{\prime }$) in buffer B


Sample minibatch of N transitions ($s,a,r,s^{\prime }$) from buffer B


Set target actor: $a^{\prime }=\pi_{\phi^{\prime }}\left(s^{\prime }\right)+\epsilon$


Calculate target value:

$y_{t}=r_{t}+\gamma {min}_{i=1,2}Q_{\theta_{i}^{\prime }}(s^{\prime },a^{\prime })$(18)

Update critics $\theta_{i}$:

$\theta_{i}\leftarrow argminN^{-1}\sum {\left(Q_{i}\left(s,a\right)-y_{t}\right)}^{2}$(19)

**If** $R<R_{d}$ **then**


Update ϕ by deterministic policy gradient:$\nabla_{\phi }J\left(\phi \right)=N^{-1}\sum {\left.\nabla_{a}Q_{\theta 1}\left(s,a\right)\right|}_{a=\pi_{\phi }\left(s\right)}\nabla_{\phi }\pi_{\phi }\left(s\right)$


Update target networks:


$\begin{array}{c} \theta_{i}^{\prime }=\kappa \theta_{i}+(1-\kappa )\theta_{i}^{\prime }\\ \phi^{\prime }=\kappa \phi +(1-\kappa )\phi^{\prime } \end{array}$(20)

**Else**


**break**

**end If**
**end For****end For**

## 4. Experiments

**Figure 4:**
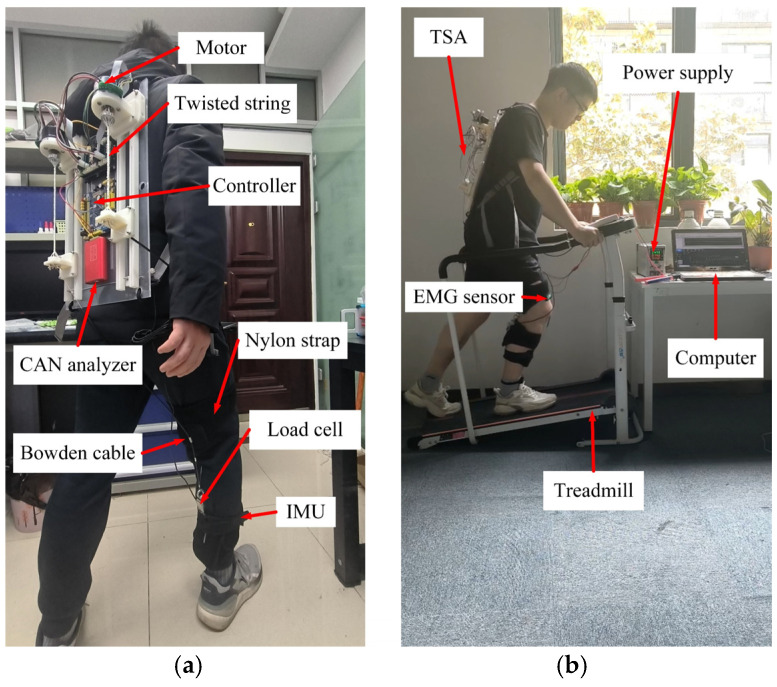
Experimental setup. (**a**) Robot prototype; (**b**) Treadmill experiment.

To validate the proposed robotic system, ten hemiplegic patients participated in an experiment with the soft exoskeleton. The motors used for the TSA are brushless DC motors (Maxon EC60 Flat), and the motors are controlled by a control board (STM32F103C8T6, STMicroelectronics, Geneva, Switzerland) after receiving the motor encoder information. In addition, a control board (NVIDIA Jetson Nano, NVIDIA Corporation, Santa Clara, CA, USA) is set to capture multi-channel sensor signals and run the RL algorithm. The real-time communication between the STM32F103C8T6 core board and Nvidia Jetson Nano board is achieved via serial communication. Inertial measurement units, force sensors, and EMG electrodes are attached to the lower limbs of the subjects to acquire multi-channel sensory information about the human-exosuit system. To ensure the accuracy of the biological signals of the human limb, the raw EMG signals are collected using a portable surface EMG acquisition system (Biometrics Ltd., WS250, UK) with a sampling rate of 2000 Hz. The band-pass (from 10 Hz to 500 Hz) and notch filters (50 Hz) are used to remove noise. The subjects are asked to walk on a treadmill while wearing the exosuit. The speed and slope of the treadmill can be modulated at their preference. The experiment setup is shown in Figure 4.

During the experiments, the subjects are asked to walk on a treadmill, and the IL tries to activate its own muscle force to complete the symmetric walking gait with respect to the HL. The patients relax their muscles between each trial. The RL-based controller is trained to optimize the exosuit assistance, and some hyperparameters are set: the parameters of the adaptive controller (12) are selected as $K_{1}=5$, $K_{2}=-20$, and $K_{3}=0.1$; the weights for each reward function term are modified with the relative entropy inverse RL algorithm [27], and they are $\Lambda_{1}=10$, $\Lambda_{2}=2$, $\Lambda_{3}=1.6$, $\Lambda_{4}=0.9$, and $\Lambda_{5}=0.5$; for the RL agent, the learning rate is κ=0.001, and the size of minibatch is 128.

**Figure 5:**
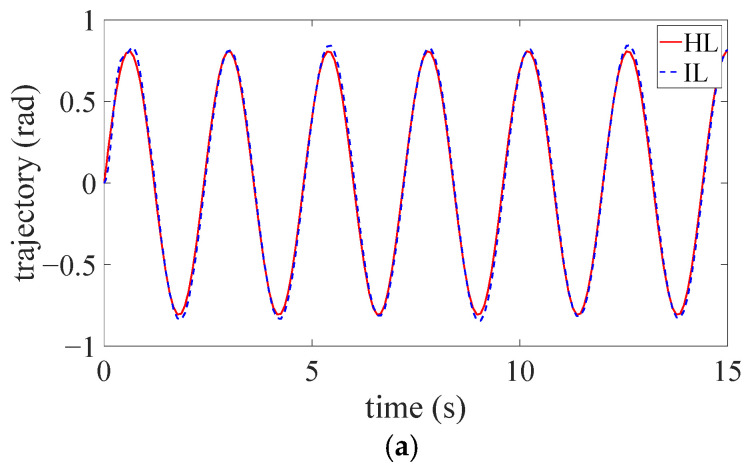

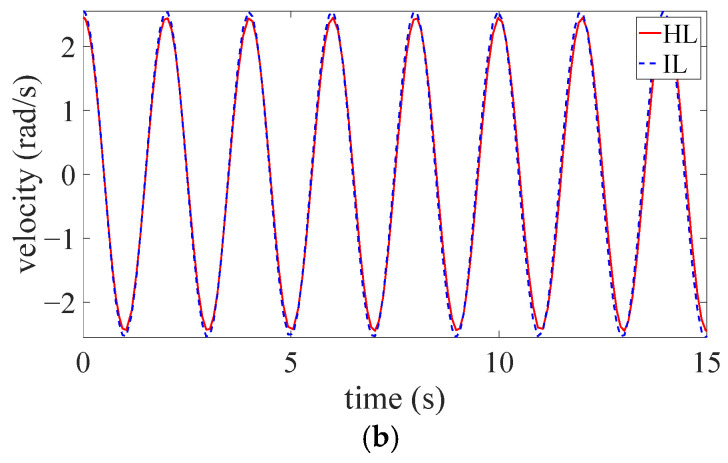
The tracking effect of the exosuit during the experiment. (**a**) The position of the desired trajectory, reference trajectory, and actual trajectory; (**b**) The velocity of the desired trajectory, reference trajectory and actual trajectory.

During the experiments, the HL’s movement is assumed as the desired trajectory for controller design, and the IL completes the prescribed walking gait with the assistance of the exosuit. Three tests are conducted to record the mean values. The HL’s trajectory position ($q_{d}$) and IL’s trajectory position ($q_{a}$) are depicted in Figure 5a, and the HL’s trajectory velocity (${\dot{q}}_{d}$) and IL’s trajectory velocity (${\dot{q}}_{a}$) are depicted in Figure 5b. It can be seen in the figures that the trajectory position and velocity tracking errors are quite small (0.06 rad and 0.11 rad/s, respectively). Due to the factors such as unmodeled uncertainties, measurement errors, and subject variance, the actual trajectory cannot perfectly track the desired trajectory. Generally, the trajectory position and velocity tracking performance are both favorable. As the human-exosuit interaction training processes, the proposed RL algorithm can achieve optimal parameters for the impedance controller to realize accurate bilateral trajectory tracking. In this regard, the user can autonomously master the motion of the impaired IL by his/her own HL, and the training safety can thus be guaranteed.

**Figure 6:**
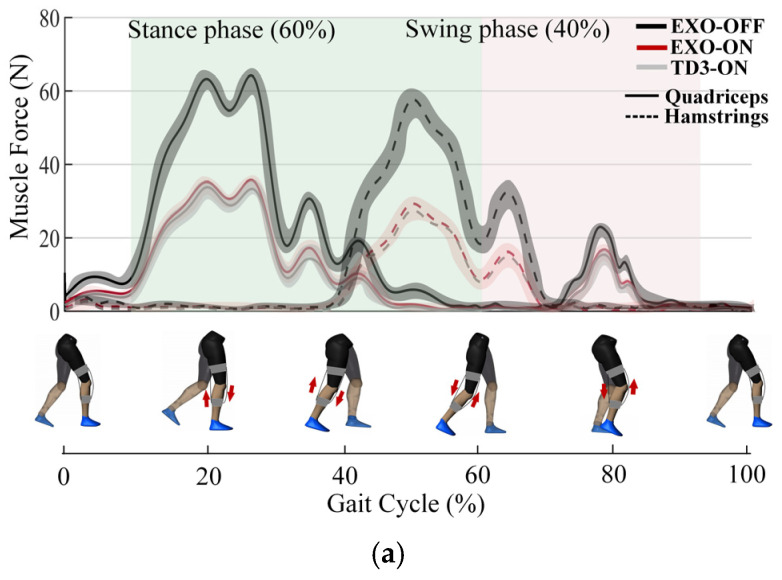

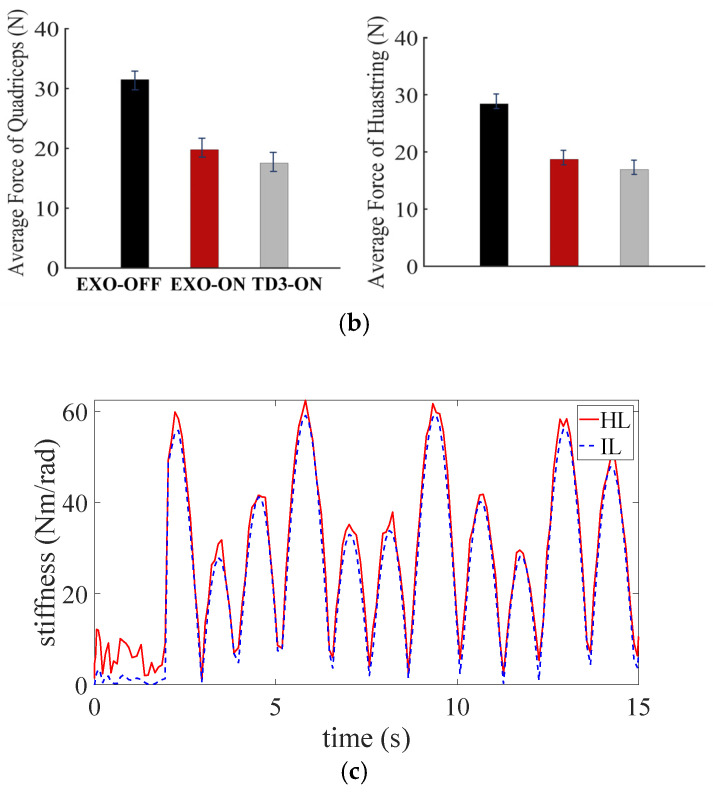
The assistance effectiveness of the exosuit. (**a**) The muscle force during a complete walking gait; (**b**) the muscle forces of quadriceps and hamstrings under the three conditions; (**c**) the stiffness profiles of the HL and IL.

Human joint impedance is vital to evaluate the performance of the proposed controller. In the proposed skill transfer strategy, in addition to the movement trajectory, the HL’s stiffness is also transferred to the IL with the assistance of the exosuit. The voluntary muscle forces of a pair of agonist and antagonist muscles, i.e., quadriceps and hamstrings, are captured by the EMG signals. The experiments are carried out under three different conditions: (a) wearing the exosuit without activating it (EXO-OFF); (b) activating the exosuit without the proposed controller (EXO-ON); (c) activating the exosuit with the proposed controller (TD3 ON). Particularly, in the EXO-ON condition, the RL algorithm is not employed by the fixed impedance parameters. Participants rested for at least 10 min before each test to reduce the effects of fatigue. The muscle forces in the three conditions are shown in Figure 6. During the stance and swing phases of a complete walking gait, the muscle force is reduced by 64.9% and 40.4% in the TD3-ON condition compared with the EXO-OFF condition, respectively. Although the muscle force saving in the EXO-ON condition is decent, the introduction of the RL algorithm can greatly enhance the assistance effectiveness. Also, the stiffness profiles are illustrated, and the tracking performance between the HL and IL is satisfactory, which indicates that the proposed skill transfer strategy can make the IL mimic the motor function of the HL with the assistance of the exosuit.

To demonstrate the superiority of the proposed controller, some state-of-the-art candidate methods are introduced to conduct comparison experiments. Some prevalent control strategies are selected as the candidate methods as follows:

***Method 1***: An impedance control is used to formulate the human-exosuit interaction, and the impedance model parameters are regulated with EMG signals according to subjective performance [29].

***Method 2***: A human-exosuit coupled dynamic model is constructed, and the individual joint impedance is optimized to accommodate various interactive tasks by emulating human central nervous system behaviors [15].

***Method 3***: An impedance control is designed, and an LQR problem is addressed with an integral RL algorithm to optimize the impedance model parameters [30].

***Method 4***: A model-free RL controller is used to receive the physical and biological information of the human-exosuit coupled system and maximize the exosuit assistance efficiency within a mirror training framework [20].

***Method 5***: A DMP-based motion generation scheme is built to plan the movement trajectory and stiffness profile, and the coupled model parameters are optimized with a RL algorithm within a mirror training framework [2].

The aforementioned methods are conducted with the same human subjects, training tasks, and robot prototype. These methods are compared in terms of trajectory tracking performance, assistance effects, and learning efficiency. Specifically, the integral of the absolute value of the errors and the integral of the square value of the errors are used to evaluate the trajectory tracking performance. The assistance effect of the exosuit is evaluated with the proportion of the IL’s muscle activation with respect to the HL’s. Learning efficiency is assessed by recording the number of the walking gaits till the reward is converged and the optimal assistance effectiveness is obtained. Generally, smaller trajectory tracking errors, lower muscle activation level, and shorter convergence time indicate better control performance. The comparison results are listed in Table 1.

In terms of tracking performance, bioinformatics-based non-learning controllers (***Method 1*** and ***Method 2***) exhibit decent trajectory tracking accuracy, but the RL-based methods have better effectiveness in modulating the robotic movement (***Proposed Method***, ***Method 3***, ***Method 4***, and ***Method 5***). In fact, non-learning controllers suffer from relatively large trajectory tracking errors due to their fixed impedance model parameters throughout the operation procedure, the complexity of constructing EMG-driven models, and uncertain parameters of the human-robot coupled model. In addition, the mirror training strategy is included in the ***Proposed Method***, ***Method 4***, and ***Method 5***, which further ensures highly accurate bilateral trajectory tracking.

In terms of assistance efficiency, ***Method 1*** reduces muscle activation by establishing a biological model, but its generalization is lacking when faced with different subjects. Many uncertain parameters exist in the human-exosuit coupled model in ***Method 2***, making it difficult to obtain precise assistance intensity to actuate the robot. ***Method 3***, ***Method 4***, and ***Method 5*** all employ RL to optimize the impedance model while responding to different subjects; however, the reward functions are identified for medical rehabilitation instead of walking assistance, and they are hard to make suitable for the soft exoskeleton where more uncertain modeling parameters are involved. Compared with ***Method 3***, the ***Proposed Method***, combining RL and biological modeling, exhibits superior universality for different subjects and targeted voluntary force saving. Compared with ***Method 4*** and ***Method 5***, the ***Proposed Method*** requires fewer weight coefficients adjustments, increasing the accuracy of determining the assistance torque of the exosuit. The concept of skill transfer is utilized in the ***Proposed Method***, ensuring that both the movement trajectory and stiffness profile are modulated according to the subjective performance, and the optimal control parameters can be determined to simultaneously realize appropriate robotic motion and stiffness. Importantly, this procedure is completely explored by the wearer him/herself with the RL-based mirror training.

By employing a specifically accommodated RL algorithm in the Proposed Method and reducing uncertain parameters in the human-exosuit coupled dynamic model and actuation controller, it enhances the model optimization efficiency, consequently reducing the learning time, compared with the learning-based methods, i.e., ***Method 3***, ***Method 4***, and ***Method 5***. As the EMG-stiffness model is utilized in advance, avoiding too many weights for each muscle EMG signal incorporated in the RL reward function is crucial.

Overall, the proposed method surpasses the advanced candidate approaches, providing excellent trajectory tracking performance, assistance effectiveness, and learning efficiency while ensuring a safe and comfortable wearing experience.

## 5. Discussion

In this article, a soft exoskeleton is developed for assisting hemiplegic patients in their daily life activities. Through the development of TSAs and proposing a bilateral skill transfer control strategy, the robotic system obtains evident advantages in many aspects. First, the high-strength and variable-stiffness actuation of the TSA are beneficial to meeting requirements of different subjects. The dynamic model of the human-exosuit coupled system is mathematically formulated, which lays foundation for the actuator stiffness regulation and human-in-the-loop controller design. Second, as the concept of the bilateral skill transfer is introduced, the movement trajectory of robot with the IL can be easily controlled to accurately track the HL’s motion. The average bilateral trajectory tracking error is less than 0.08 rad. The HL’s joint stiffness can also be transferred to the exosuit’s actuation stiffness, so that the IL is driven to complete the natural and continuous walking gait as the HL. Third, the RL algorithm is deployed to explore the optimal assistance strategy for different subjects. Complete duplication of the HL’s trajectory and stiffness to the IL side is easy with traditional impedance control but is not reasonable, because the hemiplegic patients’ lower limbs may be injured again by the inaccurate and direct changing of the robotic actuation intensity. There is a trade-off between the bilateral trajectory and stiffness tracking, and it is difficult to simultaneously realize both. The proposed RL algorithm effectively addresses this problem by setting an appropriate reward function. The above advantages are validated through the prototype experiments in terms of accurate bilateral trajectory tracking performance, highly efficient assistance, and strong adaptability to different subjects.

The limitations of the proposed system are also unavoidable. RL algorithms can be employed to pursue optimal impedance model parameters, but extravagant training processes may threaten subjects’ safety, especially for impaired patients. Computation complexity is an important issue in the current RL-based controllers. In this study, the proposed controller needs only 100 walking gaits to complete the data collection and training, and the combination of the model-based controller and the RL algorithm proves an effective way to shorten the training time. By utilizing the TD3 network architecture, the learning efficiency has been verified to outperform state-of-the-art methods through experiments. In future work, other intelligent algorithms and simulation-to-reality methods will be deployed to realize more efficient exploration of optimal strategy. In addition, the bilateral gait tracking can be easily completed for the exoskeletons on flat ground, but for some complex terrains, such as slops and stairs, the practical requirements for the exosuit wearers are not solved by mirror gait. Thus, more suitable control strategies for different environments are essential for better assistance in daily life activities. In this study, both posture and EMG sensors are used to capture the system configuration. Nonetheless, they are not portable when wearers conduct certain daily life activities and even outdoor exercise. In the latest studies, wearable sensors are applied in wearable robotics, where wearable sensors can be closely integrated with the human body. Combining wearable sensors and machine learning approaches can improve monitoring quality in training intervention, assessment, and remote monitoring [18]. The wearable sensors are also expected to be embedded in the proposed soft exoskeleton along with learning controllers. In this way, the accurate and real-time measurement of human intention and robot configuration can be achieved for better control performance. Additionally, as a robotic system that is used for hemiplegic patients, the clinic tests should be as complete as possible, and we are seeking more hospitals, doctors, and therapists for further clinic experiments and even medical applications. The generalization of the current exosuit is also limited to hemiparesis, and further exploration must be undertaken to apply the exosuit to other illnesses (like stroke, Parkinson’s disease, and spinal cord injury) and fields (like medical rehabilitation, industrial manufacturing, and military operation).

After conducting the experiments, questionnaires were issued for subjects’ feedback. Over 95% of the testers had a comfortable experience during operation. In the past, most hemiplegic patients felt embarrassed to wear traditional rigid exoskeletons, because the exoskeletons make them look different in society, and the assistance effectiveness is not consistent with their intention and capability. The proposed exosuit can be treated as clothing, which helps patients mentally accept using the robot-assisted device. The proposed controller is completely commanded by the user’s HL itself, and the contribution of the RL is self-learning the HL’s walking gait and muscle function. By wearing this robot, the subjects can find that their motor functions are recovered, particularly when they can autonomously train and improve the IL’s movement capability. It is reported by the subjects that the proposed robot system has the potential to significantly improve their life quality.

## 6. Conclusions

To assist hemiplegic patients in daily life activities, a soft exoskeleton actuated by TSAs is proposed in this paper. The structure of the exosuit is introduced, and the human-exosuit coupled dynamic model is constructed. To meet the requirements of patients with various motor functions, an adaptive impedance controller is designed to implement mirror training, where the subject can autonomously complete walking gait with a safety guarantee. The impedance model parameters are optimized with an RL algorithm, so that the HL’s physical and biological skills can be transferred to the IL with the assistance of the exosuit. Experimental validation demonstrates the effectiveness of the proposed robotic system. Comparison experiments are also conducted to manifest the advantages of the proposed controller, surpassing the state-of-the-art methods in terms of trajectory tracking performance, assistance effects, and learning efficiency.

In future work, considering the impact of the environment on human-robot interaction systems, visual sensors will be employed to effectively capture environmental information and incorporated in the vision-based controller design. In addition, more intelligent control strategies will be proposed to enhance the robotic generalization for subjects with different illnesses.

## Figures and Tables

**Table 1:** Comparison results.

Method	Integral of Absolute Tracking Errors	Integral of Squared Tracking Errors	Relative Muscle Activation	Number of Training Gaits
**Proposed**	**1.8**	**1.27**	**19.17%**	**100**
**1**	6.6	7.84	70.32%	/
**2**	4.2	6.16	32.61%	/
**3**	2.3	1.98	23.73%	160
**4**	2.8	2.35	67.21%	580
**5**	2.1	1.87	65.29%	275

## Data Availability

Data are contained within the article.
